# Total-Evidence Dating under the Fossilized Birth–Death Process

**DOI:** 10.1093/sysbio/syv080

**Published:** 2015-10-22

**Authors:** Chi Zhang, Tanja Stadler, Seraina Klopfstein, Tracy A. Heath, Fredrik Ronquist

**Affiliations:** ^1^Department of Bioinformatics and Genetics, Swedish Museum of Natural History, SE-104 05 Stockholm, Sweden;; ^2^Department of Biosystems Science and Engineering, Eidgenössische Technische Hochschule Zürich, 4053 Basel, Switzerland;; ^3^Swiss Institute of Bioinformatics (SIB), Switzerland;; ^4^Department of Invertebrates, Natural History Museum Bern, CH-3005 Bern, Switzerland;; ^5^Department of Integrative Biology, University of California, Berkeley, CA 94720 USA;; ^6^Department of Ecology and Evolutionary Biology, University of Kansas, Lawrence, KS 66045, USA;; ^7^Department of Ecology, Evolution & Organismal Biology, Iowa State University, Ames, IA 50011, USA

**Keywords:** Bayesian phylogenetic inference, birth–death process, MCMC, relaxed clock, total-evidence dating, tree prior

## Abstract

Bayesian total-evidence dating involves the simultaneous analysis of morphological data from the fossil record and morphological and sequence data from recent organisms, and it accommodates the uncertainty in the placement of fossils while dating the phylogenetic tree. Due to the flexibility of the Bayesian approach, total-evidence dating can also incorporate additional sources of information. Here, we take advantage of this and expand the analysis to include information about fossilization and sampling processes. Our work is based on the recently described fossilized birth–death (FBD) process, which has been used to model speciation, extinction, and fossilization rates that can vary over time in a piecewise manner. So far, sampling of extant and fossil taxa has been assumed to be either complete or uniformly at random, an assumption which is only valid for a minority of data sets. We therefore extend the FBD process to accommodate diversified sampling of extant taxa, which is standard practice in studies of higher-level taxa. We verify the implementation using simulations and apply it to the early radiation of Hymenoptera (wasps, ants, and bees). Previous total-evidence dating analyses of this data set were based on a simple uniform tree prior and dated the initial radiation of extant Hymenoptera to the late Carboniferous (309 Ma). The analyses using the FBD prior under diversified sampling, however, date the radiation to the Triassic and Permian (252 Ma), slightly older than the age of the oldest hymenopteran fossils. By exploring a variety of FBD model assumptions, we show that it is mainly the accommodation of diversified sampling that causes the push toward more recent divergence times. Accounting for diversified sampling thus has the potential to close the long-discussed gap between rocks and clocks. We conclude that the explicit modeling of fossilization and sampling processes can improve divergence time estimates, but only if all important model aspects, including sampling biases, are adequately addressed.

In recent years, there has been increasing interest in dated phylogenies, as they inform a wide range of questions in evolutionary biology. Advances in sequencing technologies coupled with new, statistically rigorous inference methods have greatly enhanced our ability to investigate phylogenetic relationships, but this is only the first step toward a dated phylogeny. Molecular data only provide evolutionary distances in units of evolutionary change, such as substitutions per site. Branch lengths measured in this way are the product of the geological time duration (e.g., in myr) and the evolutionary rate (e.g., in substitutions per site per myr). To estimate rates and times separately (on a relative scale), it is necessary to introduce additional model assumptions that account for branch-rate variation across the tree and the distribution of speciation events over time. Early studies achieved this by considering the evolutionary rate to be constant over time, that is, assuming a global molecular clock (also called a strict clock; Zuckerkandl and Pauling 1965). More recent methods allow the rate to vary over time under constraints specified by a relaxed-clock model, typically using a Bayesian inference framework (Hasegawa et al. 1989; Kishino et al. 1990; Thorne et al. 1998; Huelsenbeck et al. 2000; Yoder and Yang 2000; Kishino et al. 2001; Thorne and Kishino 2002; Aris-Brosou and Yang 2002; Yang and Yoder 2003; Drummond et al. 2006; Lepage et al. 2006; 2007; Rannala and Yang 2007; Drummond and Suchard 2010; Heath et al. 2012; Heath and Moore 2014).

Regardless of whether a strict or a relaxed-clock model is used, the result is an estimate of species divergence times on a relative scale. To convert the relative times into absolute times, it has been customary to rely on user-specified internal nodes that are calibrated using additional information, typically from biogeographic events or from the fossil record. Using one or more such calibration nodes, it is possible to estimate the ages of all other nodes in the tree. This calibration technique—referred to here as “node dating”—can treat the ages of the calibration nodes as known without error (Graur and Martin 2004; Hedges and Kumar 2004), or assign probability distributions to them (Thorne et al. 1998; Tavaré et al. 2002; Yang and Rannala 2006; Ho and Phillips 2009; Wilkinson et al. 2011; Heath 2012).

An alternative approach to estimating divergence times was proposed recently and has been termed “total-evidence dating” (Pyron 2011; Ronquist et al. 2012b). Compared with node dating methods, the total-evidence approach introduces a number of innovations in terms of how the fossil information is incorporated in the analysis. Specifically, homologous morphological characters are coded for fossil and extant taxa and included in a combined matrix. Age estimates are assigned to individual fossils based on the dating of the strata in which they are found. These data, together with molecular sequences sampled from extant taxa, are analyzed in an integrative framework to directly inform the inference of divergence times, while accounting for uncertainty in the placement of the fossils in the phylogeny.

One of the essential strengths of the total-evidence dating approach is that it allows the probabilistic model to be expanded to include additional sources of information that could be important in dating but have not been modeled explicitly before. Here, we exploit this to address the fossilization process and the sampling procedure, both of which potentially have a major impact on divergence time estimates. This has to be done in the context of a tree model capable of accommodating speciation, extinction, fossilization, and sampling. The early implementations of total-evidence dating (Lee et al. 2009; Pyron 2011; Ronquist et al. 2012b), in contrast, relied on simple tree priors that did not address fossilization and sampling.

The standard birth–death model used in phylogenetics, that is, the constant-rate reconstructed birth–death process, assumes that birth and death rates (or speciation and extinction rates) are constant over time and that no individuals (fossils) are sampled in the past (Kendall 1948; Nee et al. 1994; Yang and Rannala 1997; Gernhard 2008; Stadler 2009). Stadler (2010a) extended this process to account for serially sampled lineages (also see Didier et al. 2012); this extension is called the “fossilized birth–death” (FBD) process. The FBD process prior simultaneously models the speciation and extinction patterns and observations of fossils in a birth–death macroevolutionary framework. Heath et al. (2014) applied the FBD prior to estimate speciation times, assuming constant rates of birth, death, and fossil sampling, when morphological character data for extant and fossil taxa are unavailable. Their approach is more similar to node dating than total-evidence dating in that fossils are associated with specific clades a priori, and the lengths of the fossil branches are only informed by the FBD prior, not by character data. Recently, Gavryushkina et al. (2014) extended the FBD model to allow speciation, extinction, and sampling rates to change through time in a piecewise manner, as previously described for extant species phylogenies (Stadler 2011) and virus phylogenies (Stadler et al. 2013).

So far, work on the FBD process has assumed either complete or uniformly random sampling of fossils and extant taxa. Although this is convenient from a mathematical perspective, it potentially ignores an important bias in the data resulting from the common non-random choice of terminals for dating analyses. Höhna et al. (2011) showed that incorrect modeling of the sampling process can cause major problems in inferring speciation and extinction rates under standard birth–death models, especially in cases where investigators tried to maximize the taxonomic diversity in the sample. Such diversified sampling is arguably more common than random sampling in species-level data sets, and is even explicit in all studies at higher taxonomic levels. Consider a strongly asymmetric radiation like Hymenoptera, for instance, where a ladder with six or seven side branches leads to a crown group with 95% or more of the total species diversity, which numbers in the 100,000's at least. No evolutionary biologist would study the early radiation of the group without trying to include all of the basal divergence events, but the chance of hitting all of them in a uniform-random sampling scheme would be negligible.

Höhna et al. (2011) incorporated diversified sampling into the birth–death process by assuming that the investigator has succeeded in maximizing diversity, that is, has sampled exactly one extant descendant for each branch that was present at a specific time in the past (and has given rise to extant species). We take the same approach here to accommodate diversified sampling of extant taxa under the piecewise-constant FBD model. We use simulations to validate our implementation of the extended FBD model and to investigate its performance. We then apply total-evidence dating under this model to reinvestigate the early radiation of Hymenoptera, which was originally analyzed under a simple uniform tree prior (Ronquist et al. 2012b). We assess the influence of assumptions about the fossilization and sampling processes on the inferred divergence times, especially focusing on random and diversified sampling strategies.

## Theory

### The FBD Process

We use the FBD process as a macroevolutionary model describing the tree topology and node ages (Stadler 2010a; Heath et al. 2014; Gavryushkina et al. 2014). The model gives rise to extant species phylogenies with fossils. The process starts at time $t_{or}$ (time of origin or stem age) in the past with a single lineage (species). Lineages give birth to new lineages with a constant rate λ (speciation events), and die with a constant rate μ (extinction events).

**Figure 1.**
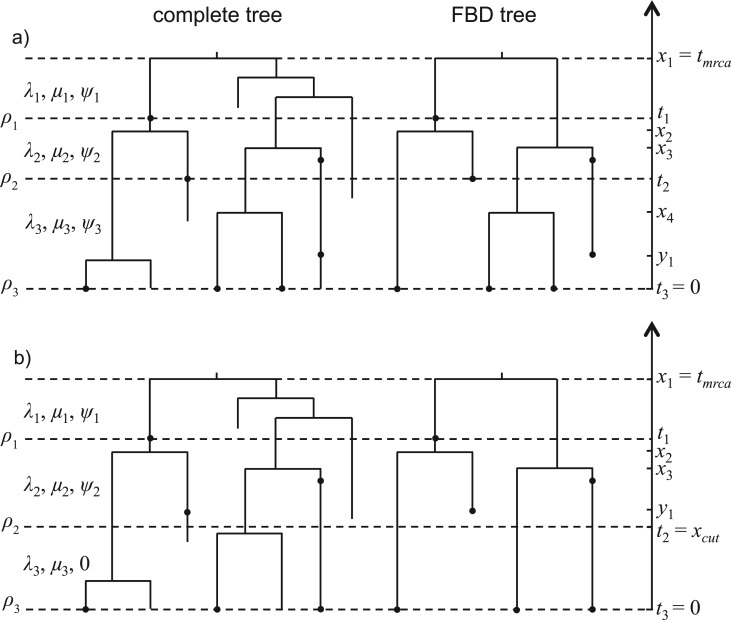
The complete tree (oriented) is generated from the birth–death process. Time from the root ($t_{mrca}$) to the present ($t_{l}=0$) is divided into l intervals by time $t_{i}$ (i=1,…,l). The birth, death, and fossil sampling rates are $\lambda_{i}$, $\mu_{i}$, and $\psi_{i}$ respectively in each interval, whereas the sampling probability at time $t_{i}$ is $\rho_{i}$. The sampled tree on the right is called FBD tree. Fossil tips not sampled at any $t_{i}$ are at time $y_{1},\ldots ,y_{m}$. Interior nodes are at time $x_{1},\ldots ,x_{m+M-1}$ with $x_{1}$ being the root age ($t_{mrca}$). There are two sampling strategies for extant taxa: a) random sampling, and b) diversified sampling.

Along branches, fossils are observed with a constant rate ψ. In addition, fossils can be observed with a constant probability ρ at pre-specified times in the past, accounting for extensive fossil sampling in particular stratigraphic layers (moments in time). The process is stopped at the present (i.e., after time $t_{or}$) when extant taxa are sampled (two sampling schemes—random and diversified sampling—are detailed below). The observed FBD tree is the tree that results when all lineages without a fossil or sampled extant descendant have been pruned away (Fig. 1). In order to distinguish between branches in the tree, we arbitrarily label the two lineages descending from each branching event with “left” or “right”; such trees are called oriented trees (Ford et al. 2009). For mathematical convenience, we derive probability densities on oriented trees.

In our implementation, we focus on trees conditioned on the crown age ($t_{mrca}$) rather than the stem age ($t_{or}$). Thus, at time $t_{mrca}$ in the past, we start with two lineages, each sharing the same time of origin (i.e, the root age). The process is further conditioned on both lineages producing sampled descendants, as otherwise the time $t_{mrca}$ would not be a crown age. In a hierarchical Bayesian model, $t_{mrca}$ would typically be considered a random variable drawn from a root age prior distribution, and the FBD distribution would then be used to calculate the probability of the rest of the tree conditional on the value of $t_{mrca}$. Unlike some other implementations of the birth–death model, we do not condition the FBD probability on the number of extant taxa.

### Modeling Parameter Shifts Over Time and Random Lineage Sampling

Similarly to Gavryushkina et al. (2014); we allow variable (piecewise constant) birth, death, and sampling rates in the complete tree (also see Stadler 2011; Stadler et al. 2013. The time from the root ($t_{mrca}$) to the present ($t_{l}=0$) is divided into l intervals by time $t_{i}$ (i=1,…,l). The birth, death, and fossil sampling rates are $\lambda_{i}$, $\mu_{i}$, and $\psi_{i}$ in interval $\left[t_{i-1},t_{i}\right)$, whereas the (uniformly random) sampling probability at time $t_{i}$ is $\rho_{i}$ (Fig. 1a).

In order to state the probability of the FBD tree under this model, we need some additional notation. We use $M_{i}$ for the number of sampled tips at time $t_{i}$ and $K_{i}$ for the number of sampled fossils with sampled descendants at time $t_{i}$. $N_{i}=M_{i}+K_{i}$ is the total number of samples at time $t_{i}$, and $n_{i}$ is the number of lineages present in the tree at time $t_{i}$ but not sampled at this time (i=1,…,l). For example, in the FBD tree of Figure 1a, $K_{1}=M_{2}=n_{1}=1$, $M_{3}=n_{2}=3$, and $n_{3}=0$. Let $M=\sum_{i=1}^{l}M_{i}$ and $K=\sum_{i=1}^{l}K_{i}$. Between $t_{i-1}$ and $t_{i}$, we use $m_{i}$ for the number of sampled fossil tips and $k_{i}$ for the number of sampled fossils with sampled descendants (i=1,…,l). Let $m=\sum_{i=1}^{l}m_{i}$ and $k=\sum_{i=1}^{l}k_{i}$. Fossil tips not occurring at time $t_{i}$ (i.e., the tips sampled with rate ψ) are at time $y_{1},\ldots ,y_{m}$. Interior branching times are at time $x_{1},\ldots ,x_{m+M-1}$ with $x_{1}$ being the root age ($t_{mrca}$).

The probability density of the FBD tree, T, conditioned on the crown age being at time $x_{1}$, a derivation analogous to that in (Stadler et al. 2013), is

(1) $$\begin{array}{l} f(T\;|t_{mrca}=x_{1},\lambda ,\mu ,\psi ,\rho ,t)=\\ \frac{q_{1}^{2}(x_{1})}{{\left(1-p_{1}(x_{1})\right)}^{2}}\prod_{i=1}^{k}\psi_{I(y_{i})}\prod_{i=2}^{m+M-1}\lambda_{I(x_{i})}q_{I(x_{i})}(x_{i})\prod_{i=1}^{m}\frac{\psi_{I(y_{i})}p_{I(y_{i})}(y_{i})}{q_{I(y_{i})}(y_{i})}\\ \prod_{i=1}^{l}{\left[(1-\rho_{i})q_{i+1}(t_{i})\right]}^{n_{i}}\rho_{i}^{N_{i}}q_{i+1}^{K_{i}}(t_{i})p_{i+1}^{M_{i}}(t_{i}), \end{array}$$

with

I(t)=i, iff ti<t≤ti−1,Ai=(λi−μi−ψi)2+4λiψi,Bi=(1−2(1−ρi)pi+1(ti))λi+μi+ψiAi,pi(t)=λi+μi+ψi−Ai1+Bi−(1−Bi)eAi(ti−t)1+Bi+(1−Bi)eAi(ti−t)2λi,qi(t)=4eAi(ti−t)(1+Bi+(1−Bi)eAi(ti−t))2,

for i=1,…,l, and $p_{l+1}(t_{l})=q_{l+1}(t_{l})=1$.

If the condition is on the stem age ($t_{or}$) instead of crown age ($t_{mrca}$) (Gavryushkina et al. 2014), then the probability density is

(2) $$\begin{array}{l} f(T|t_{or}=x_{0},\lambda ,\mu ,\psi ,\rho ,t)=\\ \frac{q_{1}(x_{0})}{1-p_{1}(x_{0})}\prod_{i=1}^{k}\psi_{I(y_{i})}\prod_{i=1}^{m+M-1}\lambda_{I(x_{i})}q_{I(x_{i})}(x_{i})\prod_{i=1}^{m}\frac{\psi_{I(y_{i})}p_{I(y_{i})}(y_{i})}{q_{I(y_{i})}(y_{i})}\\ \prod_{i=1}^{l}{\left[(1-\rho_{i})q_{i+1}(t_{i})\right]}^{n_{i}}\rho_{i}^{N_{i}}q_{i+1}^{K_{i}}(t_{i})p_{i+1}^{M_{i}}(t_{i}). \end{array}$$

The joint posterior density under the FBD prior is

(3) $$\begin{array}{l} f(T,\lambda ,\mu ,\psi ,\rho ,t,\theta |D)\propto f(D|T,\theta )\\ f(T|t,\lambda ,\mu ,\psi ,\rho )f(\lambda ,\mu ,\psi ,\rho ,t)f(\theta ). \end{array}$$

For λ, μ, ψ being constant and l=1 (no ρ sampling of fossils), this model simplifies to the model described in Stadler (2010a; Equation (5)) and Heath et al. (2014).

### Diversified Sampling of Extant Taxa

To model diversified sampling of extant taxa, we assume that exactly one representative extant species per clade descending from some cutoff time $x_{cut}$ is selected (Lambert and Stadler 2013; section “Higher-level phylogenies”) (Fig. 1b), and state the probability of such a sampled tree as $T_{x_{cut}}$. Such a sample maximizes the diversity, if diversity is measured as the total time length of the branches in the tree. We assume that the fossil sampling rate ψ is 0 in $\left[x_{cut},t_{l}\right)$, and that $\rho_{i}=0$ for $t_{i}\in [x_{cut},t_{l})$. Following the notation above, the tree $T_{x_{cut}}$ has $N_{l}$ extant sampled tips and $\eta_{l}$ extant tips not sampled, so that the overall number of extant taxa in the complete tree is $N_{l}+\eta_{l}$. For example, in Figure 1b, $N_{3}=3$ and $\eta_{3}=2$. Let lineage i ($i=1,\ldots ,N_{l}$) at time $x_{cut}$ have $\eta_{l_{i}}+1$ descendants at present, with $\sum_{i=1}^{N_{l}}\eta_{l_{i}}=\eta_{l}$. Let $p(k|x_{cut})$ be the probability that a lineage after time $x_{cut}$ has k descendants. Then, in analogy to (Stadler and Bokma 2013),

(4) $$\begin{array}{l} f(T_{x_{cut}})=f(T)\prod_{i=1}^{N_{l}}p(\eta_{l_{i}}+1|x_{cut})/p(1|x_{cut})\\ =f(T){\left(1-\frac{1}{F(x_{cut})}\right)}^{\eta_{l}}, \end{array}$$

where f(T) is the probability density of the tree on $N_{l}$ tips assuming complete sampling (i.e., using Equation (1) with $\rho_{l}=1$), and where the last equality follows from Lambert and Stadler (2013; Equation (1)).

F(t) is calculated as follows (Smrckova and Stadler, personal communication). For t in $\left[t_{i-1},t_{i}\right)$, and denoting t by $t_{i-1}$, we have

(5) $$F(t)=1+\sum_{k=l}^{i}G(t_{k}),$$

with

(6) $$G(t_{k})=\frac{\lambda_{k}}{\lambda_{k}-\mu_{k}}\left(e^{\left(\lambda_{k}-\mu_{k})(t_{k-1}-t_{k}\right)}-1\right)e^{\sum_{j=k+1}^{l}\left(\lambda_{j}-\mu_{j})(t_{j-1}-t_{j}\right)}.$$

Note that for l=k, we have $e^{\sum_{j=l+1}^{l}\left(\lambda_{j}-\mu_{j})(t_{j-1}-t_{j}\right)}=1$.

For λ and μ being constant in $\left[x_{cut},t_{l}\right)$ ($t_{l}=0$) and $t_{l-1}=x_{cut}$, then $F(x_{cut})=1+G(t_{l})$, and

(7) $$f(T_{x_{cut}})=f(T){\left(\frac{\lambda_{l}(1-e^{-(\lambda_{l}-\mu_{l})t_{l-1}})}{\lambda_{l}-\mu_{l}e^{-(\lambda_{l}-\mu_{l})t_{l-1}}}\right)}^{\eta_{l}}.$$

For inference, we typically consider trees where the samples (i.e., nodes corresponding to observed fossils or extant taxa) are labeled, instead of oriented trees. To convert an oriented tree into a labeled tree, we multiply the probability density of an oriented FBD tree (i.e., Equation (1) or (7)) with $2^{\left(m+M-1\right)}/(m+M+k+K)!$. Rather than sampling $\lambda_{i}$, $\mu_{i}$, and $\psi_{i}$, we operate on $d_{i}=\lambda_{i}-\mu_{i}$ (net diversification), $r_{i}=\mu_{i}/\lambda_{i}$ (turnover), $s_{i}=\psi_{i}/(\mu_{i}+\psi_{i})$ (fossil sampling proportion) (i=1,…,l). With the sampling probabilities (the ρ values), there are potentially 4l parameters in the full FBD model (Fig. 1). However, it is usually not possible to estimate the sampling probabilities of a birth–death model independently of the speciation and extinction rates (Stadler 2012). In the empirical analyses, we fix the sampling probability of extant taxa ($\rho_{l}$) to a value based on estimates of the current species diversity. Further, we set all other ρ values to zero and instead use the fossilization rate $\psi_{i}$ in each interval i to model the product of fossilization rate and fossil sampling probability in that time interval. We thus only estimate 3l parameters in our analyses. When assuming constant birth, death, and fossilization rates (l=1), the number of parameters reduces to three.

### MCMC Proposals for FBD Trees

**Figure 2.**
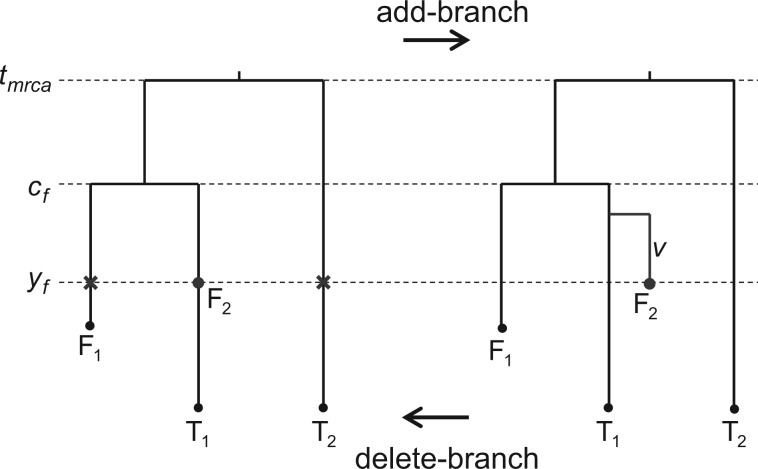
$F_{1}$ and $F_{2}$ are two fossils, $T_{1}$ and $T_{2}$ are extant taxa. $F_{2}$ is either split from the lineage of $T_{1}$ using the add-branch move (left to right) or merged to the lineage of $T_{1}$ using the delete-branch move (right to left). The two crosses on lineages of $F_{1}$ and $T_{2}$ at time $y_{f}$ represent two alternative positions to which we could move fossil $F_{2}$ using a SPR move.

To allow Markov chain Monte Carlo (MCMC) sampling of the FBD process model, we implemented two reversible-jump Markov chain Monte Carlo (rjMCMC) proposals, the “add-branch” and “delete-branch” moves of Heath et al. (2014) (see also Lewis et al. 2005), in MrBayes (Ronquist et al. 2012a). The add-branch move selects an ancestral fossil and then adds a speciation event and a branch leading to that fossil, effectively changing the ancestral fossil to one that represents a sampled extinct lineage (Fig. 2). The delete-branch move positions a tip fossil on its sibling lineage by deleting the branch leading to it (Fig. 2). It is not allowed if the tip fossil is younger than its sibling. The two moves are chosen with equal probability, but we abort the add-branch move if there is no ancestral fossil, or abort the delete-branch move if there is no fossil tip. For example, on the left tree of Figure 2, we have one fossil tip ($F_{1}$) and one fossil ancestor ($F_{2}$), whereas on the right tree, obtained after an add-branch move, both fossils are tips. The Hastings ratios and Jacobian factors for each move are detailed below ($k^{\prime }$ is the number of fossil ancestors, and $m^{\prime }$ is the number of fossil tips in the tree).

#### Add-branch move

Choose an ancestral fossil with probability $1/k^{\prime }$. Choose a branch length for the fossil from a uniform distribution on (0, $c_{f}-y_{f}$), where $c_{f}$ is the age of the ancestral node of the fossil and $y_{f}$ is the age of the fossil. For the corresponding delete-branch move, choose a tip fossil (probability $\frac{1}{m^{\prime }+1}$) and put it on the sister branch without changing the age of the fossil. The Hastings ratio is $\frac{k^{\prime }}{m^{\prime }+1}$ and the Jacobian is $c_{f}-y_{f}$.

#### Delete-branch move

Choose a tip fossil with probability $1/m^{\prime }$. Put the fossil on the sister branch without changing its age. For the corresponding add-branch move, choose an ancestral fossil (probability $\frac{1}{k^{\prime }+1}$) and obtain the length of its subtending branch from a uniform distribution on (0, $c_{f}-y_{f}$), where $c_{f}$ is the age of the ancestral node of the fossil and $y_{f}$ is the age of the fossil. The Hastings ratio is $\frac{m^{\prime }}{k^{\prime }+1}$, and the Jacobian is $\frac{1}{c_{f}-y_{f}}$.

We also extended two topology proposals, the node-slider and the subtree-pruning-and-regrafting (SPR) moves, to update the position of ancestral fossils on the FBD tree. These proposals do not change the number of branches. The node-slider move changes the position of an ancestral fossil along the branch on which it sits using a sliding window with reflection. This move does not change the topology, and its Hastings ratio is 1. For example, in the tree to the left, $F_{2}$ can be moved along the subtending branch of $T_{1}$ with the upper boundary of min($c_{f}$, $u_{f}$) and the lower boundary $l_{f}$, where $u_{f}$ and $l_{f}$ are the upper and lower ages of fossil $F_{2}$ (Fig. 2).

The SPR proposal can move an ancestral fossil to another branch without changing its age (Fig. 2). One of the two candidate branches crossing time $y_{f}$ (lineage $F_{1}$ and $T_{2}$) is chosen with equal probabilities for attaching $F_{2}$ at time $y_{f}$. The Hastings ratio is 1. Note that the nearest-neighbor-interchange (NNI) move is a special case of SPR. In this example, moving $F_{2}$ to the branch subtending $F_{1}$ is a NNI move.

## Simulations

We performed two rounds of simulations, primarily to verify our implementation of the FBD priors (see also Supplementary Table S3 on Dryad at http://dx.doi.org/10.5061/dryad.26820 for empirical validation of our implementation against BEAST2 (Bouckaert et al. 2014; Gavryushkina et al. 2014), using models available in both softwares). Therefore, we focus on scenarios where the fossils provide a substantial amount of dating information. However, we also look at the behavior of the model in various situations where there is a slight mismatch between the generating model and the model used for inference.

### Simulation I: No Sequence Data

The TreeSim package in R (R Development Core Team 2009; Stadler 2010b) was used to simulate a complete tree under the constant-rate birth–death process with birth rate λ = 0.3, death rate μ = 0.2, and 100 extant taxa. We then sampled extant taxa and fossils in the complete tree with one of the following four strategies:

1. Sample extant taxa with probability ρ = 0.5, and sample fossils with a constant rate ψ = 0.1.
2. Sample extant taxa with probability $\rho_{2}$ = 0.5, and sample fossils with a constant rate ψ = 0.1. One additional time for sampling fossils is set at $t_{1}$ = 10 in the past, where each lineage is sampled with probability $\rho_{1}$ = 0.5.
3. Sample extant taxa with proportion 0.5 to maximize diversity ($x_{cut}=t_{1}$ is adjusted to sample half of the extant taxa, and $\rho_{1}=0$), and sample fossils with a constant rate $\psi_{1}$ = 0.1 prior to $t_{1}$, and $\psi_{2}=0$ in (0, $t_{1}$).
4. Sample extant taxa with proportion 0.5 to maximize diversity ($x_{cut}=t_{2}$ is adjusted to sample half of the extant taxa, and $\rho_{2}=0$), and sample fossils with a constant rate $\psi_{1}=\psi_{2}$ = 0.1, and $\psi_{3}=0$ in (0, $t_{2}$). At $t_{1}$ = 10 ($>t_{2}$), fossils are sampled with probability $\rho_{1}$ = 0.5.

For each generated FBD tree, we first fixed the tree topology and branch lengths, and ran MCMC without sequence data to sample net diversification (d=λ−μ), turnover (r=μ/λ), and fossil sampling proportion (s=ψ/(μ+ψ)) from the FBD prior in MrBayes. Four chains (one cold, three heated) were run for 1 million iterations and sampled every 200 iterations, with the first 25% samples discarded as burn-in. The following hyperpriors were used for the parameters: d∼ Exponential(10) (we use the rate parameterization for the exponential distribution throughout the article, unless noted otherwise), r∼ Beta(1, 1), s∼ Beta(1, 1), and $\rho_{i}$'s were fixed to their true values. We generated 1000 replicates for each sampling strategy (4000 FBD trees in total).

We then added the tree proposals described above to also sample the FBD trees from the prior (without sequence data). In these runs, the prior for $t_{mrca}$ and for the fossil ages were fixed to their true values, and the chain length was enlarged to 2 million.

**Figure 3.**
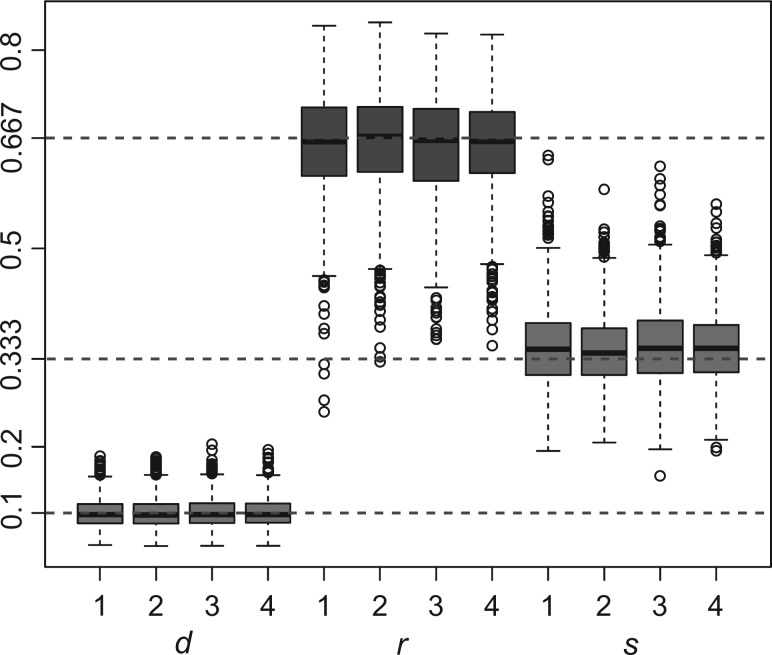
Box-plot of the posterior means of d=λ−μ, r=μ/λ, and s=ψ/(μ+ψ) for 1000 replicates under strategy 1 to 4 in simulation I (fixed trees). The true values of d, r, and s are 0.1, 0.667, and 0.333, respectively (horizontal lines).

The mean value of the posterior estimates from these runs is very close to the corresponding true value in each case when the trees were fixed (Fig. 3). The coverage probabilities for the parameters are high when we also sampled the trees from the priors (Table 1). Thus, these results indicate that our implementation of the FBD prior-density calculations and MCMC proposals is correct.

**Table 1.** Coverage probabilities of parameter estimates in analyses of trees from Simulation I (no sequence data)

	Coverage Prob.			
Prior	d	r	s	% Ancestral
I.1	0.97	0.84	0.86	0.87
I.2	0.95	0.85	0.83	0.83
I.3	0.91	0.87	0.84	0.89
I.4	0.94	0.90	0.89	0.91

Notes: The coverage probabilities (rate of the true value falling into the 95% HPD interval) for d=λ−μ, r=μ/λ, s=ψ/(μ+ψ), and the proportion of ancestral fossils are summarized for each prior setting. I.x stands for Simulation I prior x (see Simulation section for details). FBD model parameters d, r, and s were constant through time in both the generating model and the inference model.

### Simulation II: Trees Inferred from Sequence Data

We picked 100 trees randomly from the 1000 trees generated using strategy 2 and strategy 4 in Simulation I. We simulated DNA sequences on each tree using Seq-gen (Rambaut and Grass 1997) under the JC69 model (Jukes and Cantor 1969). The mutation rate was arbitrarily set to 0.003 (each branch length of the FBD tree was multiplied by this rate). To put this in a real-time perspective, if one time unit is assumed to correspond to 10 myr, then time 10 means 100 Ma, and the mutation rate would be 0.03% per site per myr. If one time unit is assumed to be shorter, then the corresponding absolute rate would increase proportionally, and vice versa. The sequence length was set to 500 bp.

We then used MCMC sampling in MrBayes to infer the node ages (root age in particular), net diversification (d=λ−μ), turnover (r=μ/λ), and fossil sampling proportion (s=ψ/(μ+ψ)) from the sequence data, alongside the tree. The following priors were used for the parameters: d∼ Exponential(10), r∼ Beta(1, 1), s∼ Beta(1, 1), and $t_{mrca}∼$ Uniform (0, 1000). The evolutionary model was set to JC69, the true model. The prior for the global clock rate was set to a Gamma(3, 1000) distribution, which has a mean equal to the true clock rate.

We also investigated the impact of different prior assumptions concerning the sampling process. For alignments generated from trees in strategy 2 (random sampling of extant taxa), the following prior parameters were used:

1. Random sampling of extant taxa with probability 0.5, extra fossil sampling at time 10 with probability 0.5 (denoted T for “true model”).
2. Random sampling of extant taxa with probability 0.5, no extra fossil sampling at time 10 (denoted C for “constant rate”).
3. Larger sampling fraction (probability 1.0, i.e., complete sampling) of extant taxa, no extra fossil sampling at time 10 (denoted L for “larger sampling fraction”).
4. Diversified sampling of extant taxa with proportion 0.5, no extra fossil sampling at time 10 (denoted D for “different sampling strategy”).
5. Uniform tree prior (denoted U for “uniform tree prior”).

For alignments generated from trees in strategy 4 (diversified sampling), the same prior settings were used (runs 1′ – 4′), except that we used diversified sampling for 1′ (T) and 2′ (C), and random sampling for 4′ (D). Note that the uniform tree prior (U) (Ronquist et al. 2012b), which posits a uniform prior density on speciation times given the fossil and root age constraints, does not include parameters specific to the FBD model, that is, diversification, turnover, and fossil sampling rates.

For priors 1 and 1′ (piecewise-constant rates), we inferred the rate parameters (d, r, s) before and after time 10 separately, and accounted for the sampling effort at time 10 and 0 (present). For these cases, the prior models used in MCMC inference were thus consistent with the simulation settings (the true model), except for the uncoupling of the rate parameters (d, r, s) in the two time intervals. For the other FBD priors, we assumed constant diversification, turnover and fossil sampling rates and ignored the extra fossil sampling effort at time 10, causing various types of misfit between the prior (the model assumed in inference) and the model used in the simulation (the true model). Under diversified sampling, the cutoff time, $x_{cut}$, was adjusted to be slightly younger than the youngest internal node and also the youngest fossil. The fossil ages were fixed to the true values used in the simulation. The tree topology and branch lengths were not fixed but inferred from the simulated sequence data using MCMC sampling, employing the add-branch and delete-branch moves among others. The MCMC chains (one cold, three heated) were run for 2 million iterations and sampled every 200 iterations, with the first 25% samples discarded as burn-in.

**Figure 4.**
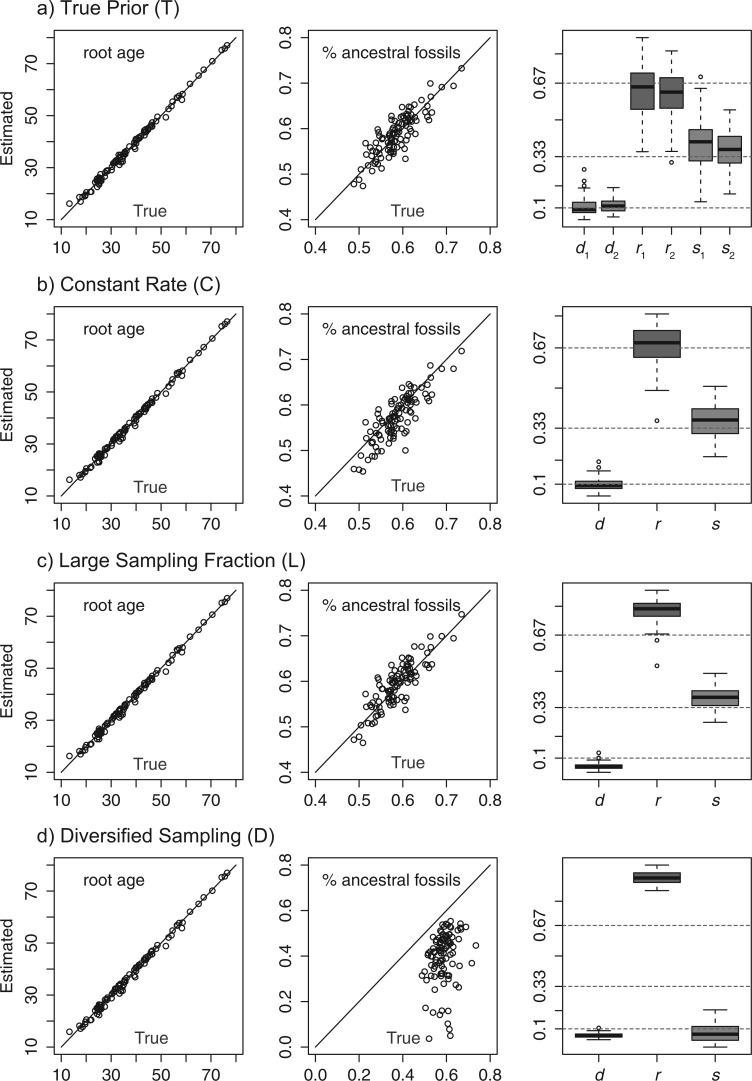
The posterior means of the root age and proportion of ancestral fossils against their true values, and box-plot of the posterior means of d, r, and s for the four scenarios in Simulation II. The true strategy is random sampling of extant taxa.

**Figure 5.**
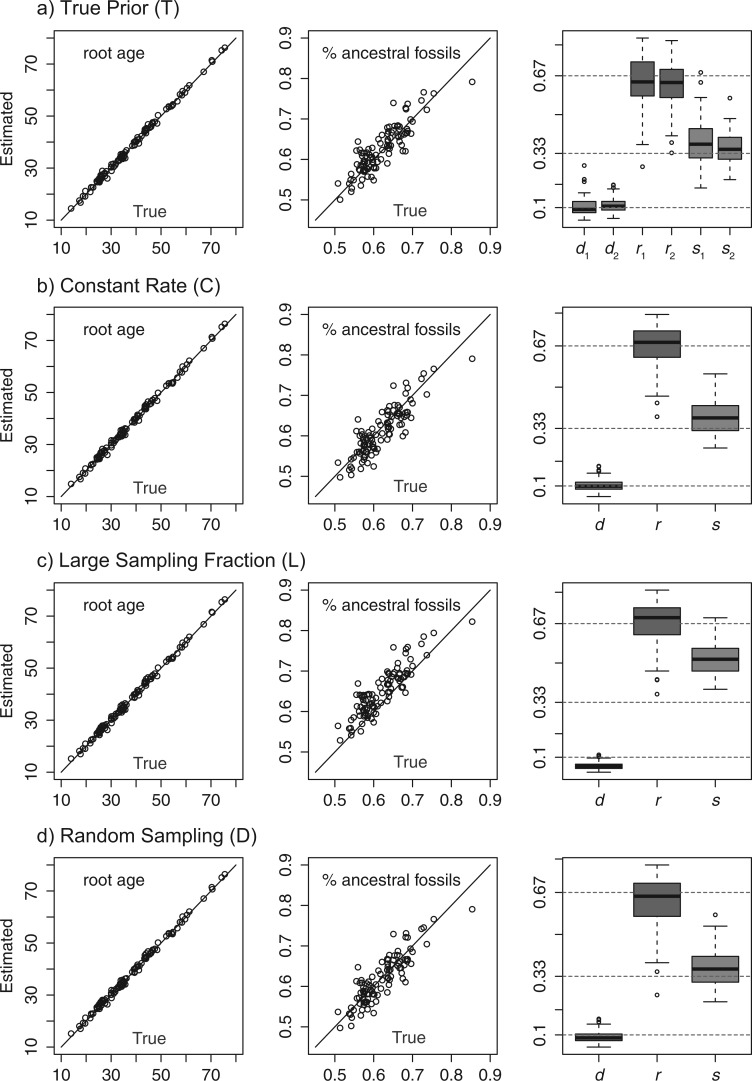
The posterior means of the root age and proportion of ancestral fossils against their true values, and box-plot of the posterior means of d, r, and s for the four scenarios in Simulation II. The true strategy is diversified sampling of extant taxa.

The results show that when the FBD prior is consistent with the simulation settings (II.1 and II.1′), the estimates are close to the true values, with coverage probabilities for the root age being 0.88, and >0.9 for the other parameters (Table 2, Figs. 4 and 5, T). When assuming no extra fossil sampling at time 10, the accuracy of those estimates is almost the same as in the previous case (Table 2, C). When assuming a larger sampling fraction of extant taxa (complete sampling), the rate estimates can be biased, causing the underlying parameters λ, μ, and ψ to also be biased (Table 2, L).

**Table 2.** Coverage probabilities of parameter estimates in analyses of data from Simulation II (trees inferred from sequences)

	Coverage Prob.				
Prior	Root Age	d	r	s	% Ancestral
FBD trees from Simulation I.2 (random sampling)					
II.1 (T)	0.88	0.93, 0.94	0.96, 0.94	0.93, 0.94	0.97
II.2 (C)	0.88	0.96	0.95	0.95	0.93
II.3 (L)	0.86	0.51	0.66	0.91	0.94
II.4 (D)	0.84	0.79	0.00	0.00	0.04
II.5 (U)	0.46	—	—	—	—
FBD trees from Simulation I.4 (diversified sampling)					
II.1′ (T)	0.88	0.94, 0.93	0.96, 0.93	0.95, 0.96	0.89
II.2′ (C)	0.87	0.95	0.94	0.93	0.92
II.3′ (L)	0.84	0.43	0.94	0.24	0.70
II.4′ (D)	0.85	0.89	0.93	0.93	0.93
II.5′ (U)	0.48	—	—	—	—

Notes: The coverage probabilities (rate of the true value falling into the 95% HPD interval) for the root age ($t_{mrca}$), d=λ−μ, r=μ/λ, s=ψ/(μ+ψ), and the proportion of ancestral fossils are summarized for each prior setting. II.x stands for Simulation II prior x (see Simulation section for details). FBD model parameters d, r, and s were inferred separately for the two time intervals in 1 (T) and 1′ (T); therefore, two values are given for these. In the other analyses (C, L, D), a single value was assumed for both time intervals. There are no birth, death, or sampling parameters for the uniform tree prior (U).

Somewhat surprisingly, the estimates are poor under diversified sampling when the true strategy is random sampling (Fig. 4, D), but accurate under random sampling when the truth is diversified sampling (Fig. 5, D) (see also Table 2). This is probably explained by the asymmetry between these scenarios. Given the same fossil sampling rate, there are more fossils recovered under random sampling than under diversified sampling of extant taxa because we assume that no fossils are sampled after the cutoff time in the latter case. Thus, the data from random sampling are more informative and conflict strongly with the prior model assuming diversified sampling in 4 (D). In contrast, the data from diversified sampling are less informative and therefore conflict less strongly with the prior model in 4′ (D). We note that the accuracy of the root age estimate is almost the same in the four cases (T, C, S, D) (Table 2, Figs. 4 and 5), even when the prior is biased so that the other estimates may be biased. This is important as the focus in dating analyses is the inference of absolute node ages.

Under the uniform prior, the posterior mean of the root age is usually smaller than the true value. Although the absolute difference between the inferred and true values is small, the credibility interval is narrow so that the coverage rate is low (∼0.5) (Table 2). Apparently, the fact that the uniform prior does not model ancestral fossils causes a slight but consistent bias toward a younger estimate of the root age when many fossils are actually ancestral. One can possibly explain this as an effect of the different visibility of multiple substitutions along certain branches under these scenarios. An ancestral fossil can reveal that evolution took a more complicated route than is immediately apparent, resulting in longer branch-length estimates and deeper divergence times. When the same fossil sits on a side branch, which is always the case in the uniform tree prior, such complicated routes can be simplified by pushing apparently unique changes onto the side branch and thus shortening the estimated length of the trunk of the tree. This might be the cause of the underestimation of the root age under the uniform tree prior when there are ancestral fossils in the data.

We conclude by noting that our simulated data are highly informative about divergence times, as the fossil sampling is rate-constant and rich, the fossils have complete sequences with known fixed ages, and there is no rate variation across the tree. These settings are appropriate for validating the algorithms, and they may fit empirical scenarios where we have access to an abundance of well-preserved fossils or ancient DNA, and when there is little rate variation across the tree. However, divergence time estimates are likely to be less robust under more realistic settings, and additional simulations exploring the performance of FBD-based inferences in these cases would be valuable. In particular, when the fossil sampling is stratigraphic and the fossils are poorly preserved, as is the rule for empirical data sets, the FBD prior might have considerable impact on the divergence-time estimates (see Hymenoptera analysis below) so that it becomes important to model the fossilization and sampling processes appropriately. Furthermore, rate variation across the tree can cause both loss of accuracy and problems with inference biases, especially if the rate variation is not properly addressed by a relaxed-clock model. Finally, under realistic data-poor scenarios, there may also be problems associated with overparameterization (overfitting).

## Total-evidence dating of hymenoptera

To investigate the performance of the FBD model in an empirical setting, we reanalyzed a data set on the early radiation of Hymenoptera (Ronquist et al. 2012b). This data set includes 60 extant and 45 fossil Hymenoptera and eight outgroup taxa (Table 3; see also Tables 1 and 2 in Ronquist et al. (2012b)). The data were divided into eight partitions as follows: (i) morphology, (ii) 12S and 16S, (iii) 18S, (iv) 28S, (v) 1st and 2nd codon positions of CO1, (vi) 3rd codon position of CO1 (but not used in our analyses), (vii) 1st and 2nd codon positions of both copies of EF1α (Klopfstein and Ronquist 2013), and (viii) 3rd codon position of both copies of EF1α. As in Ronquist et al. (2012b), we used the Mk model (Lewis 2001) with the “variable” ascertainment bias and gamma-distributed rate variation across characters for the morphology partition. For the molecular partitions, we used the general time-reversible model with gamma rate variation (GTR + Γ), except for 28S where we employed the SYM model with equal base frequencies and symmetric exchange rates (SYM + Γ). Parameters of the substitution models and among-site rate variation were unlinked across partitions, and partition-specific rate multipliers were used to account for variation of evolutionary rates across partitions. Specifically, a flat Dirichlet prior was used for the relative partition rate multipliers (the partition rate multiplier times the proportion of sites in that partition). The relative partition rate multipliers were constrained to sum to 1.0, so that the average partition rate multiplier was 1.0 across sites (characters) in the data set.

**Table 3.** Summary of Hymenoptera data

Taxa	Time (Ma)	Number
Fossils	235 (228, 242)	2
	187.5 (174, 201)	1
	182.5 (182, 183)	4
	179.5 (168, 191)	4
	164.5 (161, 168)	3
	157.5 (152, 163)	17
	148.5 (145, 152)	1
	138.5 (125, 152)	1
	135 (125, 145)	6
	134 (133, 135)	2
	119 (113, 125)	2
	97 (94, 100)	1
	83 (80, 86)	1
Hymenoptera	0	60
Outgroups	0	8
Sum		113

Notes: The data include 60 extant and 45 fossil hymenopteran taxa and 8 outgroups. The estimated fossil ages are given as the midpoint and the time interval of the corresponding geographical stratum. The bounds of the stratum were used as parameters to a uniform prior on the fossil age in the total-evidence dating analyses.

In contrast to the previous analysis (Ronquist et al. 2012b), which treated fossil ages as known without error, here we accounted for the uncertainty in those estimates. The fossil ages were assigned uniform prior distributions with ranges corresponding to the age ranges of the respective strata (Table 3).

We used both the uniform tree prior (Ronquist et al. 2012a), as in the previous analysis, and different versions of the FBD prior. For the FBD prior, we first assumed constant birth and death rates. For random sampling of extant taxa, the fossil sampling rate was assumed to be constant through time, whereas for diversified sampling, fossil sampling was constant (nonzero) before the cutoff time ($x_{cut}$) and zero thereafter. The value of $x_{cut}$ was adjusted during the MCMC so that the cutoff was just after the youngest internal node (and the youngest fossil) in the FBD tree.

Because all fossils included in our data set were sampled from the Mesozoic (Triassic, Jurassic, and Cretaceous) and none from younger or older strata, we also used piecewise-constant rates in the FBD prior. For random sampling, the two rate-shifting times were fixed to 252 Ma (separating the Permian and the Triassic) and 66 Ma (separating the Cretaceous and the Paleocene). The birth, death, and sampling rates shift at the same time, so that we inferred three rates each for speciation, extinction, and fossil sampling, respectively. For diversified sampling, we only used one rate-shifting time for birth and death rates at 252 Ma, because the cutoff time ($x_{cut}$, the additional shifting time for the fossil sampling rate) is very close to or slightly older than 66 Ma (see results below). To infer separate birth and death rates after time $x_{cut}$ would lead to very uncertain estimates, because that time interval by definition consists of single lineages leading to each extant taxon. The fossil sampling rate after time $x_{cut}$ is zero by definition. Under this approach, we thus inferred only two rates each for speciation, extinction, and fossil sampling, respectively.

We used an Exponential(100) prior for net diversification (d), and a Beta(1, 1) prior for turnover (r) and fossil sampling proportion (s). The sampling probability (or sampling proportion) for the extant taxa was set to 0.0005; this estimate is based on the number of described extant hymenopteran species (Aguiar et al. 2013). The prior for the root age ($t_{mrca}$) was set to “offsetExp(315, 396)”, that is, an offset exponential distribution with mean 396 (oldest insect fossil) and minimum 315 (oldest neopteran fossil). The holometabolan taxa were constrained to be monophyletic, and the prior for their age was set to “offsetExp(302, 396)” with minimum 302 (age of the oldest putative Holometabola). The holometabolan constraint are enforced to root the tree properly, as the clock model itself is not sufficient to provide the correct rooting for this data (Ronquist et al. 2012a).

We used a lognormal(−7.1, 0.5) prior for the substitution rate (the base rate of the clock) with mean 0.0009, median 0.0008, and mode 0.0006. These settings were chosen by comparing the age of the oldest insect fossil with the root age estimation from uncalibrated clock analyses as discussed in Ronquist et al. (2012a). We performed sensitivity analyses to determine the robustness of our estimates to changes in the priors on root age and clock rate (see results below). Ronquist et al. (2012a) noted that different relaxed-clock models produced very different divergence time estimates for this data. To explore whether these differences would be affected by replacing the simplistic uniform tree prior with the more realistic FBD model, we used two relaxed-clock models: the autocorrelated lognormal rates (TK02) model (Thorne and Kishino 2002) with an Exponential(0.2) prior for the variance increase parameter, and the uncorrelated independent gamma rates or “white noise” (IGR) model (Lepage et al. 2007) with an Exponential(37) prior for the variance increase parameter. As currently implemented in MrBayes, the compound Poisson process (CPP) model (Huelsenbeck et al. 2000) is computationally not compatible with the FBD model, and was not included in this study.

We executed four independent runs in parallel for each analysis, each consisting of four Metropolis-coupled MCMC chains (one cold, three heated). The length of each run was initially set to 50 million iterations, but enlarged to 100 million for the piecewise-constant FBD priors to achieve better convergence. Convergence was assessed by the MrBayes built-in diagnostics: the average standard deviation of split frequencies (ASDSF; target value 0.05) and the estimated effective sample size (ESS; target value 100). We also examined trace plots of likelihoods and parameter samples. The chain was sampled every 1000 iterations, with the first 25% (or 50% for the longer chains) samples discarded as burn-in. The MCMC samples from the four parallel runs were then combined.

### Results

We first checked the induced tree prior by running the MCMC without data (by setting the sequence and morphology data likelihood to 1), under the model priors described above with fixed clock rate (0.001). We also explored a more relaxed (Exponential(1)) and a more constrained prior (Exponential(10000)) on net diversification (d). It turns out that the induced divergence time priors vary widely under different prior assumptions (Table 4). Under the uniform tree model, the mean of the root age in the induced prior is much older than 396, the expectation of the root age prior. Under the FBD model, the induced divergence times vary widely: they are sensitive both to the model details and to the prior for d. The expected proportion of ancestral fossils is consistently close to zero in the induced priors except for two cases involving the same type of FBD model (Table 4, pcFBD\_Rnd). In both these cases, the induced prior favors young trees, thus apparently increasing the probability of fossils being ancestors. This phenomenon seems to be coupled with a high net diversification rate in the induced prior, as the expected proportion of ancestral fossils decreased dramatically when the prior was focused on small values of d.

**Table 4.** Induced prior distribution on root age ($t_{mrca}$) and age of Hymenoptera

Tree Prior	Prior Root Age	Prior Age of Hymenoptera (Ma)	Proportion of Ancestral Fossils
Uniform	486.6 (395.9, 650.3)	482.4 (392.2, 648.5)	—
d∼ Exp(1)			
FBD (Rnd)	1235 (920.1, 1532.4)	1194 (893.6, 1476.9)	0.00 (0.0, 0.009)
FBD (Div)	321.6 (315.0, 343.3)	265.1 (222.8, 316.2)	0.00 (0.00, 0.01)
pcFBD (Rnd)	346.7 (315.0, 477.2)	170.4 (54.35, 288.7)	0.51 (0.14, 0.82)
pcFBD (Div)	350.9 (315.0, 484.9)	239.5 (196.4, 315.6)	0.00 (0.00, 0.02)
d∼ Exp(100)			
FBD (Rnd)	861.8 (315.0, 1483.)	832.2 (25.4, 1436.2)	0.00 (0.0, 0.009)
FBD (Div)	321.7 (315.0, 343.1)	265.3 (223.2, 315.6)	0.00 (0.00, 0.02)
pcFBD (Rnd)	355.9 (315.0, 494.3)	209.3 (61.69, 322.4)	0.40 (0.07, 0.78)
pcFBD (Div)	358.8 (315.0, 517.0)	233.5 (202.7, 252.0)	0.00 (0.00, 0.03)
d∼ Exp(10000)			
FBD (Rnd)	326.5 (315.0, 365.5)	279.2 (64.86, 345.0)	0.00 (0.00, 0.01)
FBD (Div)	320.0 (315.0, 335.7)	235.8 (196.0, 286.0)	0.00 (0.00, 0.02)
pcFBD (Rnd)	570.2 (315.0, 1098.)	458.3 (235.0, 1025.)	0.00 (0.00, 0.01)
pcFBD (Div)	360.4 (315.0, 519.6)	246.0 (219.9, 252.0)	0.00 (0.00, 0.01)

Notes: Numbers represent the median and the 95% HPD interval. The hyperprior for d=λ−μ is Exponential (rate parameter specified in the table), for r=μ/λ and s=ψ/(μ+ψ) is Beta(1, 1), for the root age is Exponential with mean 396 and offset 315, and for the fossils are uniform on the time interval of the stratum in which they were found (Table 3). For the full empirical analysis (Table 5), the intermediate prior on d was used.

**Table 5.** Posterior distribution on root age ($t_{mrca}$) and age of Hymenoptera

Tree Prior	Posterior Root Age (Ma)	Posterior Age of Hymenoptera (Ma)	Proportion of Ancestral Fossils
IGR relaxed clock			
Uniform (U)	345.1 (315.0, 402.2)	306.0 (289.3, 341.3)	—
FBD (Rnd)	406.4 (328.1, 500.3)	346.6 (291.9, 426.5)	0.00 (0.00, 0.02)
FBD (Div)	322.7 (315.1, 342.6)	279.4 (255.1, 304.3)	0.02 (0.00, 0.07)
pcFBD (Rnd)	369.6 (315.6, 433.3)	325.6 (282.5, 374.9)	0.33 (0.20, 0.47)
pcFBD (Div)	328.2 (315.0, 358.9)	251.7 (237.9, 310.9)	0.02 (0.00, 0.07)
TK02 relaxed clock			
Uniform (U)	462.5 (368.3, 549.8)	372.5 (310.1, 446.8)	—
FBD (Rnd)	523.1 (438.2, 605.4)	402.9 (344.7, 457.0)	0.00 (0.00, 0.00)
FBD (Div)	435.1 (371.3, 497.5)	343.5 (301.5, 385.0)	0.00 (0.00, 0.00)
pcFBD (Rnd)	484.1 (411.2, 551.5)	365.3 (324.3, 413.1)	0.02 (0.00, 0.07)
pcFBD (Div)	447.4 (324.9, 564.5)	346.1 (265.4, 442.7)	0.00 (0.00, 0.00)

Notes: Numbers represent the median and 95% HPD interval of the estimated posterior distribution under the uniform tree prior (U), constant-rate FBD prior with random (Rnd) or diversified (Div) sampling, and piecewise-constant FBD prior (pcFBD) with random (Rnd) or diversified (Div) sampling, and under either the IGR or the TK02 relaxed-clock model.

The full empirical analyses with the sequence and morphology data only used the intermediate Exponential(100) prior on d. The posterior estimates of root age and age of Hymenoptera in these analyses are summarized in Table 5 for a range of analyses under two kinds of relaxed-clock models (IGR and TK02) and two kinds of tree priors (uniform and FBD). In the case of the FBD prior, we assumed that sampling of extant taxa was either random or diversified.

Under the uniform tree prior (Table 5 U, see also Supplementary Figure S1 on Dryad at http://dx.doi.org/10.5061/dryad.26820), accounting for the uncertainties of fossil ages using uniform priors, the node age estimates are very similar to those in the previous study (Ronquist et al. 2012a; Fig. 9) in which the fossil ages were treated as known without error. Under the IGR model, the median age of Hymenoptera is inferred to be in the Carboniferous, with the 95% HPD stretching into the Permian. The estimates are much older under the TK02 model, with the median age of Hymenoptera dating back to the Devonian, and the 95% HPD spanning the Carboniferous, Devonian, and Silurian. The same pattern was observed in Ronquist et al. (2012b). The reason for the difference is apparently that the IGR model allows occasional extreme rate changes on adjacent branches, which makes it easier for this model to accommodate the obviously rapid rate shifts close to the root of the hymenopteran tree. The autocorrelated TK02 model has a rate smoothing effect on adjacent branches, which tends to produce longer trees. As discussed in Ronquist et al. (2012b), several lines of evidence suggest that the IGR model fits the data better. Therefore, we will focus on the IGR model in the following.

For the FBD priors, we first describe the results obtained under the assumption of constant speciation, extinction, and fossilization rates (Table 5 FBD, see also Supplementary Figures S2 and S3 on Dryad at http://dx.doi.org/10.5061/dryad.26820). Under these conditions, the modeling of the sampling process has a dramatic impact on the inferred divergence times. When extant taxa are assumed to have been sampled at random, the inferred age of Hymenoptera is older than under the uniform prior (347 Ma versus 306 Ma) and has a wider HPD interval. When changing from random to diversified sampling, the age of Hymenoptera becomes younger (279 Ma), now lying in the Permian. Compared with the random sampling prior, the diversified sampling assumption has the effect of stretching more recent and shrinking more ancestral branches, resulting in younger age estimates near the root of the tree (e.g., compare Supplementary Figures S2 and S3 on Dryad at http://dx.doi.org/10.5061/dryad.26820).

**Figure 6.**
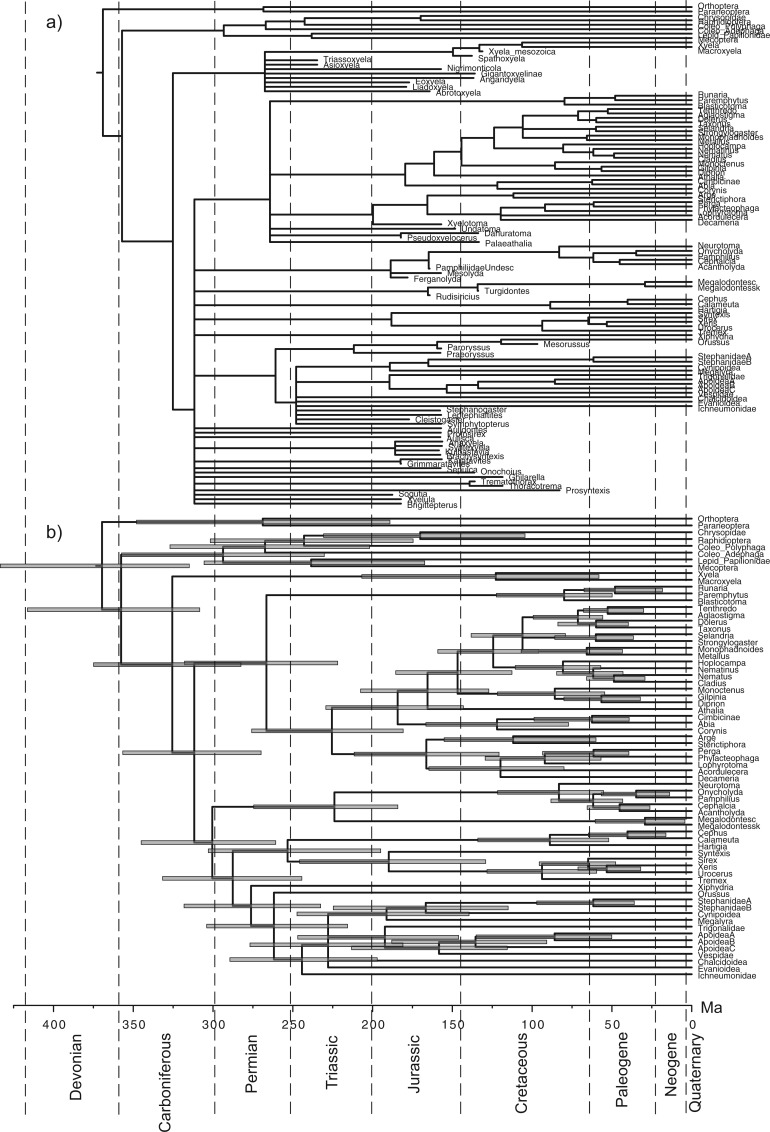
Majority-rule consensus tree, a) including all fossils and b) including only extant taxa, from total-evidence dating analysis under the piecewise-constant FBD prior with random sampling and under the IGR relaxed-clock model. Node bars indicate HPD intervals of estimated divergence times (cf. Table 5, pcFBD_Rnd).

**Figure 7.**
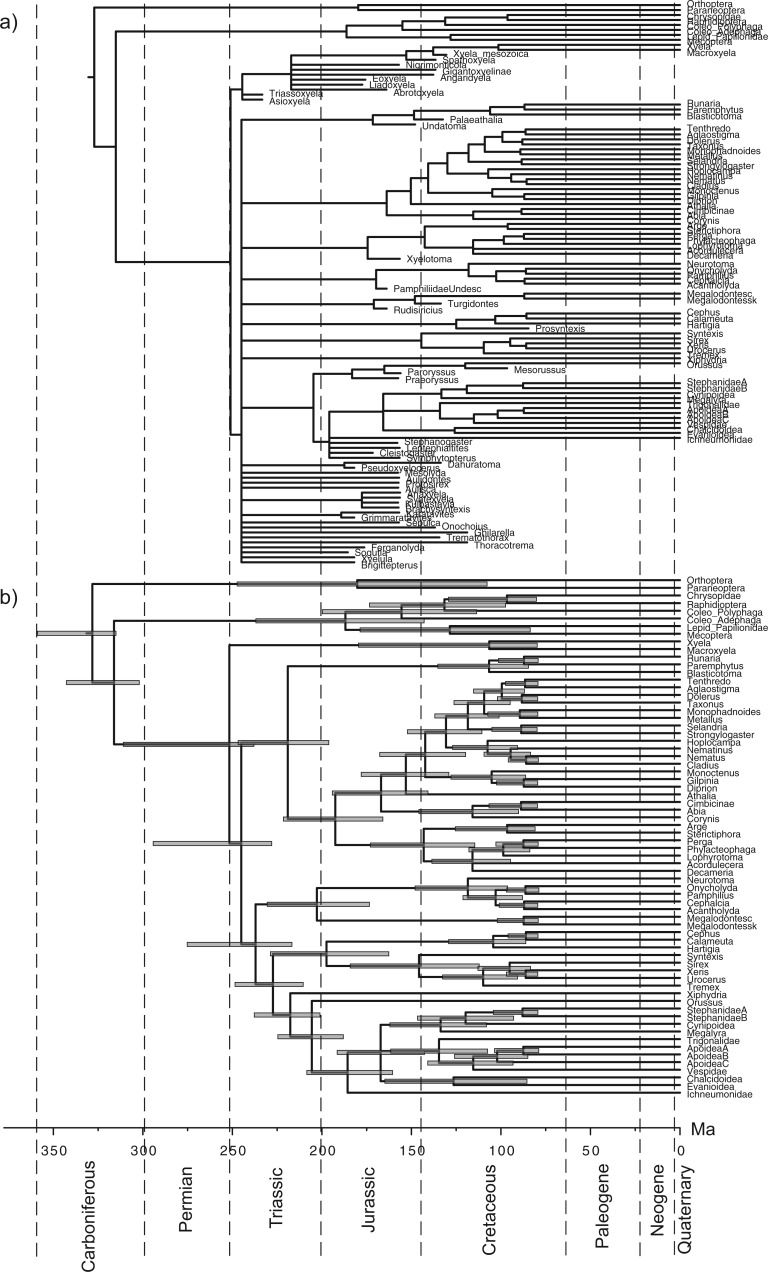
Majority-rule consensus tree, a) including all fossils and b) including only extant taxa, from total-evidence dating analysis under the piecewise-constant FBD prior with diversified sampling and under the IGR relaxed-clock model. Node bars indicate HPD intervals of estimated divergence times (cf. Table 5, pcFBD_Div).

Finally, we applied the piecewise-constant FBD priors to account for variable rates of fossil sampling. We allowed net diversification d and turnover r to shift at the same time as s at 252 Ma. For random sampling, d, r, and s were allowed to change again at 66 Ma; for diversified sampling, only s changed to zero at the cutoff time of about 70 Ma. Consequently, all fossils are included in the second time interval. The effect of allowing diversification, turnover, and fossilization rates to vary over time is to decrease the age estimates of Hymenoptera, by ∼20 myr (Table 5, Figs. 6 and 7). The estimate of 252 Ma is just at the geological time separating the Triassic and Permian. It is slightly older than the age of the oldest hymenopteran fossils, *Triassoxyela* and *Asioxyela*, which are dated to 228–242 Ma (Fig. 7).

**Figure 8.**
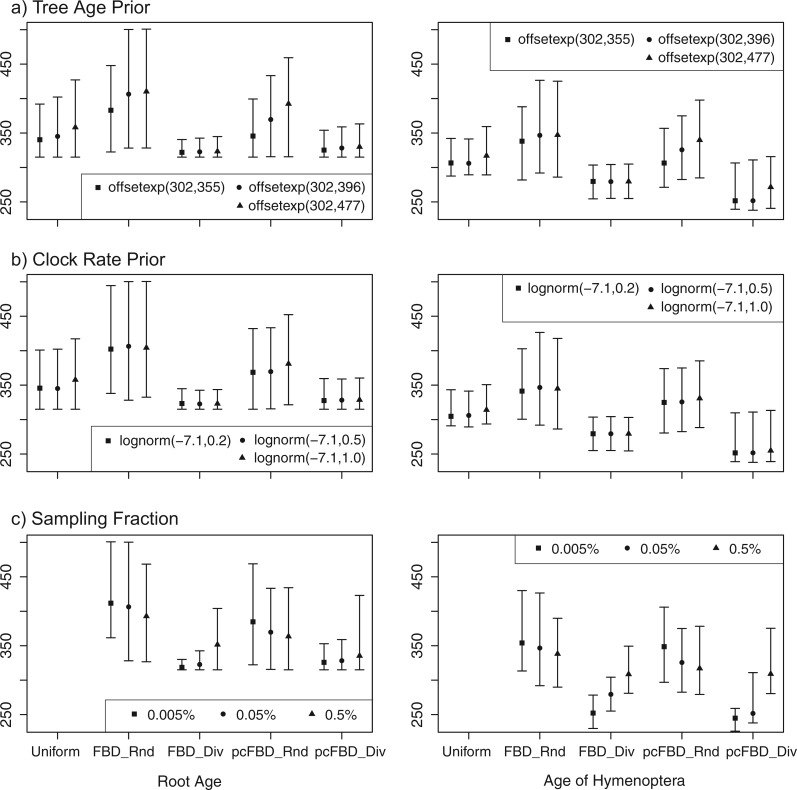
The impact of a) tree age prior, b) clock rate prior, and c) sampling fraction on posterior ages of the root and of Hymenoptera under the uniform tree prior (if applicable), FBD with random sampling (FBD_Rnd) or diversified sampling (FBD_Div), and piecewise-constant FBD with random sampling (pcFBD_Rnd) or diversified sampling (pcFBD_Div).

We examined the impact of the priors on root age and clock rate, and that of the assumed sampling fraction. In the previous analyses, the prior for the root age was an offset exponential distribution with mean 396 Ma and offset 315 Ma. We halved and doubled the distance from the mean to the minimum to obtain more and less restrictive priors. The more restrictive root-age prior had an expectation of 355 Ma, and the less restrictive prior an expectation of 477 Ma. The calibration prior for the holometabolan age was also changed so that the expectation was the same as for the root. The results show that the impact of the root calibration on the divergence-time estimates is rather small (Fig. 8a). We also used a smaller (0.2) and larger variance (1.0) in the lognormal prior for the base rate of the clock. As shown in Figure 8b, the posterior age estimates are not sensitive to the variance parameter in the clock-rate prior.

**Figure 9.**
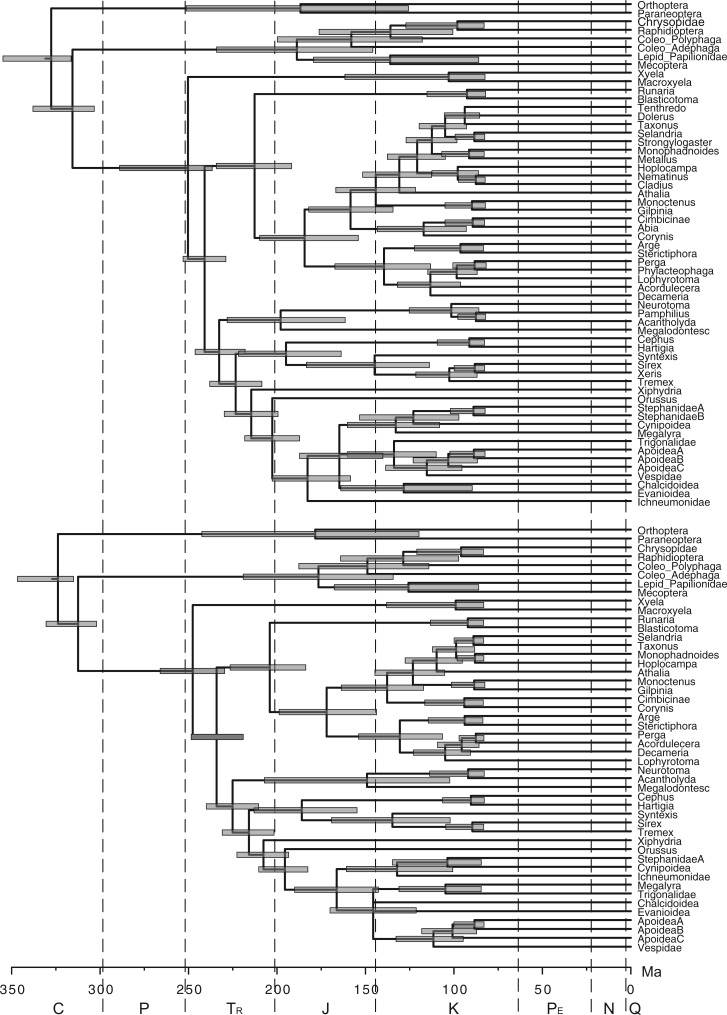
Majority-rule consensus tree of extant taxa from total-evidence dating analysis under the IGR model and the piecewise-constant FBD prior with diversified sampling. The tree above has 9 taxa removed from the original data, whereas the tree below has 20 taxa removed, to eliminate young splits that might be inconsistent with the diversified sampling model.

The sampling probability of extant taxa was fixed to 0.0005 in the previous analyses, an estimate based on a compilation of the number of currently described hymenopteran species (Aguiar et al. 2013). To assess the sensitivity of the results to changes in the assumed sampling fraction, we enlarged and reduced it by an order of magnitude (to 0.005 and 0.00005, respectively). For random sampling of extant taxa, increasing the sampling probability decreases the posterior ages slightly, whereas it increases the posterior ages considerably for diversified sampling (Fig. 8c). Eventually, the age estimates will approach those under complete sampling. These results contrast with our simulations, in which the sampling probability had only a minor effect on the age estimates (Table 2, Figs. 4 and 5). However, the sampling fractions examined in the empirical analyses are several orders of magnitude smaller than those studied in the simulations.

In the empirical analyses, almost all fossils are inferred to be tips rather than ancestral nodes in the tree (Table 5, see Supplementary Tables S1 and S2 on Dryad at http://dx.doi.org/10.5061/dryad.26820 for the estimated probability of each fossil being ancestral). The only exception involves the piecewise-constant FBD prior with random sampling under the IGR model, where approximately one-third of the fossils were estimated to be ancestral. This is the same model where the induced prior puts a high probability on fossils being ancestors (Table 4).

The consensus trees including all fossils are partially unresolved, regardless of the tree prior (Figs. 6 and 7, and Supplementary Figures S1 to S10 on Dryad at http://dx.doi.org/10.5061/dryad.26820). However, when the fossils are excluded from the sampled trees to compute the consensus, the consensus trees are fully resolved. Thus, the polytomies are entirely due to uncertainty in the placement of the fossils (see also Ronquist et al. 2012b). MCMC convergence was slightly slower for the piecewise-constant FBD prior than for the uniform tree prior or the constant-rate FBD prior. This is especially true for the topology parameter. Nevertheless, all model parameters appeared to have converged after 100 million iterations according to the trace plots, and judging from the ASDSF (<0.05), ESS (≥100), and PSRF values.

## Discussion

### Modeling Fossilization and Sampling

In this article, we examined the importance of incorporating information about speciation, extinction, and sampling of fossil and extant taxa in total-evidence dating. Clearly, these processes influence our prior beliefs concerning the shape of the tree and the placement of fossils therein. For instance, if the fossilization rate is high and the extinction rate is low, we expect most fossils to sit on branches leading to extant taxa. Conversely, if the fossilization rate is low and the extinction rate high, most fossils are instead likely to represent extinct side branches. The sampling of extant taxa also affects our prior expectations concerning the structure of the tree. For instance, if we sample extant taxa so that the chosen exemplars span as much diversity as possible (a diversified sample) (Höhna et al. 2011), then the tree should be ‘`bush-like” with long terminal branches. If extant taxa are chosen randomly instead, the branching events should be more evenly distributed over time.

Our expectations concerning the structure of the tree are also influenced by differences over time in speciation, extinction, and fossilization rates. The proportion of fossils included from a particular time horizon is strongly affected by decisions about fossil sampling, for example, which sites to explore in the search for fossils, and which fossil specimens to select for analysis. If the fossilization rate itself varies over time, this will cause some strata to be richer in fossils than others and thus better represented in the tree. Similarly, variations in speciation and extinction rates over time will cause the density of branching events in the observed tree to vary over time.

Together, all these phenomena affect the shape and structure of the tree and therefore potentially influence the estimation of divergence times. However, the tree priors used previously in total-evidence dating (e.g., Pyron 2011; Ronquist et al. 2012b; Wood et al. 2013; Dornburg et al. 2015; Arcila et al. 2015; Grimm et al. 2015) do not account for them at all. To explore their influence in total-evidence dating, the recent FBD process is an obvious choice (Stadler 2010a; Heath et al. 2014; Gavryushkina et al. 2014). The formulation of the FBD model allows us to examine the effects of speciation, extinction and fossilization processes, and to accommodate different sampling strategies. Even though birth–death models like the FBD process have their limitations, they provide a starting point in the search of more realistic tree priors.

In comparing the performance of the FBD model to previous models used in total-evidence dating, it might first seem like an obvious choice to use Bayes-factor tests, a standard Bayesian approach to model choice. Unfortunately, Bayes factors have severe limitations in this context. First, the models have different parameters and different dimensionality, which means that the outcome of a Bayes-factor test is decided to a large extent by the priors and not necessarily by model performance. The sensitivity of Bayes-factor tests to priors is illustrated well by the apparent contradictions that may arise in the testing of topology hypotheses (Bergsten et al. 2013). Second, even if it were possible to find suitable priors for appropriate Bayes-factor tests, it is difficult to estimate the Bayes factors accurately enough to allow reliable comparison of the complex models used in total-evidence dating. For instance, Ronquist et al. (2012b) tried to distinguish three different relaxed-clock models for total-evidence dating analyses using the state-of-the-art stepping-stone sampling algorithm (Xie et al. 2011) to estimate the marginal model likelihoods needed for computing Bayes factors. Despite considerable computational effort, however, the variance in the estimated marginal likelihoods between independent runs of the algorithm was larger than the differences between the models, making it impossible to distinguish them.

A pragmatic approach in these situations is to compare models with respect to their ability to account for the data. The comparison is simplified if we are comparing models defined on the same or similar parameter spaces, which is arguably the case here. At first sight, the FBD model might seem fundamentally different in that it allows fossils to represent both side branches and ancestors of other taxa in the tree, while previous models considered all fossils to be side branches (tip fossils). However, although ancestral fossils appear distinct from tip fossils, it is possible to consider them simply as the boundary case of tip fossils, when the length of the side branch goes to zero. In fact, we take this approach in our computational machinery, where ancestral fossils in the FBD model sit on side branches with length zero. Viewed in this way, the FBD model just specifies a different prior probability distribution on the same tree space used by the simpler models.

Given this similarity between the models, it is relatively straightforward to compare their ability to account for the data. At one extreme, the data could be so informative that they were able to pull the simpler tree-model priors toward the same posterior obtained under the FBD model. If so, there would only be minor differences in divergence-time estimates between models. In other words, the FBD model would not add much to the analysis except that we might be able to estimate some additional parameters of interest, like the fossilization rate. At the other extreme, the data might carry very little information about the shape of the tree, in which case we would essentially retrieve the prior under each model. The interesting case occurs when the data fit the FBD model better, but are not informative enough to modify the simple tree model priors toward the posterior observed under the FBD model. This may result in significant improvements in divergence-time estimation under the FBD model. Our results suggest that this is indeed the case we are facing. In particular, the fact that we observe informative posterior estimates of FBD model parameters indicates that the model is picking up relevant signal in the data, and thus fits the data better than previous tree priors (e.g., comparing Table 4 with 5). It also suggests that the FBD models examined here are not overparameterized; if they were, the posterior distributions would be more similar to the induced prior distributions.

Our results also show that the specific details of the FBD prior can have a strong influence on divergence-time estimates. Notably, it seems that the most important factor is how the sampling of extant taxa is modeled (Table 5). Under the assumption of random sampling, the FBD model gives results that are older than those of the uniform model. When we accommodate the fact that the sample is diversified, however, the results change substantially. Under the IGR model, which appears to be the better of the two relaxed-clock models examined here, the estimate of the crown age of Hymenoptera shifts from around 325 Ma to 252 Ma when diversified sampling is accounted for. A similar shift toward younger ages also occurs under the TK02 relaxed-clock model.

Beyond the sampling issue, our results confirm that the relaxed-clock model significantly affects the divergence-time estimates. The introduction of the FBD model did not affect the difference between the two relaxed-clock models, IGR and TK02. However, there are several reasons to prefer the IGR results over the TK02 results (Ronquist et al. 2012a). Unlike the IGR model, TK02 assumes autocorrelated evolutionary rates. Such rates are not expected to shift very rapidly, and TK02 therefore has difficulties accommodating the apparently rapid shifts in evolutionary rates close to the root of the hymenopteran tree (Ronquist et al. 2012a). This results in longer branches close to the root of the hymenopteran subtree, and is associated with substantially older divergence time estimates for the crown age of Hymenoptera. Given that the oldest known Hymenoptera fossils are around 235 Ma, the TK02 estimates appear unrealistically old (Table 5, TK02 vs. IGR), further strengthening the conclusion that the model does not fit the data well.

It is interesting to note that the result under the piecewise-constant FBD model with random sampling and IGR rates suggests that one third of the fossils are ancestors (Table 5). This proportion decreases to 2% or less under all other models, which is more in line with expectations. If one third of the fossils were indeed ancestors of extant lineages, then the diversity of Hymenoptera must have been very low during their early diversification and a large fraction of the ancient lineages must have left current descendants, both of which seem unlikely. The high proportion of inferred ancestors under this particular model is apparently due to a combination of two factors. First, the model puts considerably more prior probability on fossils being ancestral than any of the other models (Table 4). Second, the scarcity of character data for the fossils is not enough to pull the posterior away from this prior. Fossil data were only available for 4–20% of the morphological characters, depending on the completeness of the specimens (Supplementary Tables S1 and S2 on Dryad at http://dx.doi.org/10.5061/dryad.26820), and molecular data were completely missing for the fossils. Thus, it is not surprising that the inferred proportion of ancestral fossils is influenced to a large extent by the tree prior.

### Implications for the Age of Hymenoptera

The previous total-evidence dating analysis of the early radiation of Hymenoptera suggested that the extant taxa started to diversify about 309 Ma, almost immediately after splitting off from the remainder of Holometabola in the Carboniferous (Ronquist et al. 2012b). In contrast, paleontological reconstructions indicated a time lag of at least 75 myr from the origin of the order in the Carboniferous to the first separation of extant lineages in the Triassic (Rasnitsyn 1988; 2010. The results under the diversified sampling FBD model (Fig. 7) essentially confirm the paleontological reconstructions, suggesting that the previous total-evidence analysis was biased by an unrealistic tree prior. In fact, the diversified sampling results are rather close to the dates suggested by Rasnitsyn in his attempt to fit the fossil record to the hymenopteran phylogeny, minimizing the extent of “ghost lineages” (lineages undocumented in the fossil record) (Rasnitsyn 2010). For instance, Rasnitsyn suggested that crown Tenthredinoidea date back to 140 Ma (223 Ma in our analysis), Pamphilioidea to 180 Ma (202 Ma in our analysis), and Apocrita to 180 Ma (187 Ma in our analysis).

Given the obvious gaps in the early fossil record of hymenopterans, with some lineages having sister groups that are absent for 100 myr or more (Rasnitsyn 2010), the 252 Ma estimate may appear surprisingly close to the age of the oldest known hymenopteran fossils. Is it really plausible that hymenopterans started to diversify only just before we observe the first evidence of their presence in the fossil record? There is actually some reason to suspect that the age estimates obtained under the diversified FBD process may be biased toward the recent because of imperfections in the model. Consider that, for mathematical convenience, our model of diversified sampling assumes that the investigator is able to find the sample representing the maximum amount of phylogenetic diversity given the chosen number of tips. In practice, however, the investigator is likely to miss a few of the oldest splits in the tree, and instead include a few splits that are younger than the ideal cutoff value, perhaps much younger.

To investigate whether such a bias could influence our analyses, we tried to reduce the effect by eliminating the speciation-time outliers from the tree. Specifically, we inferred an uncalibrated relaxed-clock tree of extant taxa under the uniform tree prior and IGR model. The priors for the root age and the substitution rate were unchanged. We first removed nine taxa representing extremely young splits from the tree. We then eliminated eleven additional taxa representing moderately young splits, resulting in the removal of twenty extant taxa in total (Supplementary Figure S12 on Dryad at http://dx.doi.org/10.5061/dryad.26820). In the latter case, almost all splits expected to be younger than the youngest fossil (83 Ma) were eliminated. We then repeated the dating analysis under the piecewise-constant FBD model with diversified sampling. The estimated dates were very similar to those of the original analysis of the complete tree, especially for the deepest splits in the tree (Fig. 9, see also Fig. 7). This indicates that the biasing effect of the strict maximum-diversity assumption is negligible, at least for the older divergence times in the tree. Nevertheless, this may not always be the case, and relaxing the maximum-diversity assumption would appear to be an important area for further improvement of total-evidence dating under the FBD prior; indeed, for any type of dating under a birth–death prior assuming diversified sampling.

A recent phylogenomic study of insects (Misof et al. 2014) placed the earliest hymenopteran radiation in the Triassic, which is consistent with Rasnitsyn (1988; 2010) and with our results under the FBD model with diversified sampling. However, despite the impressive amounts of sequence data, the divergence-time estimates from this phylogenomic study are not necessarily more accurate, and cannot be taken as reliable confirmation of our results. This is because sequence data primarily inform the estimation of evolutionary branch lengths, while the major sources of dating uncertainty stem from the placement of calibration fossils and from the modeling of processes such as fossilization, sampling of recent and fossil taxa, and rate variation across the tree. Although the diversified FBD analysis presented here addresses all of these sources of uncertainty, the phylogenomic study was based on traditional node dating with narrow calibration priors (Misof et al. 2014), which potentially compromised the results. In addition, the dating of the hymenopteran part of the tree is limited by the sparse sampling of lineages from this order. For instance, no member of Xyelidae was included in the analysis, even though they are the sister group of all other extant hymenopteran lineages.

To further improve dating of the early hymenopteran radiation, it is important to focus on the major remaining sources of uncertainty, and address them in a total-evidence dating framework. More sequence data can help expand the number of extant terminals, increase the precision of evolutionary branch-length estimates, and possibly improve the modeling of speciation, extinction, and rate variation across the tree. However, much of the dating uncertainty will remain unless we become better at incorporating the information from the fossil record. For instance, a better understanding of morphological evolution in Hymenoptera will help place the fossils with more certainty in the phylogeny of extant taxa.

Most importantly, increasing the number of fossils included in the analysis can help elucidate the speciation, extinction, and fossil sampling processes, all of which can contribute strongly to divergence-time estimation. Our study has only scratched the surface with respect to fossils that could be informative about the early evolution of Hymenoptera, and the same could be said for many other dating studies. Some of the fossils that were not analyzed by us are poorly preserved, but we have shown earlier that even very incomplete fossils can contribute to the precision of divergence-time estimates in a total-evidence dating analysis (Ronquist et al. 2012b). Another possibility for improvement is to incorporate more stratigraphic information in the analysis, such as data on the variation in fossil sampling intensities over time. For example, the absence of insects that clearly belong to the hymenopteran crown group from strata older than the Triassic is potentially informative about the age of the crown group, yet we have not addressed this simple fact appropriately even in the most sophisticated models we explored here. To do so, we would have had to extend the sampling of fossils to the entire Holometabola and to older strata.

### Conclusions

Ronquist et al. (2012a) demonstrated that total-evidence dating can use more information from the fossil record, may avoid biases caused by erroneous placement of fossils, and can improve the precision and presumably the accuracy of divergence-time estimates. With this study, we have shown that expanding the total-evidence analysis to include information about speciation, extinction, fossilization, and sampling can result in further improvements. In particular, modeling the sampling strategy of extant taxa in a realistic way appears to have a substantial impact on divergence-time estimates. Diversified sampling, which arguably is the rule rather than the exception in dating studies, results in trees with long terminal branches and most speciation times clustered close to the root of the tree. Such trees have low prior probability under most tree priors, including FBD models assuming complete or random sampling. If extant taxa have been sampled to maximize diversity, but this is not accounted for in the model, the low prior probability of “bush-like” trees may cause significant biases in divergence-time estimates, in particular, unrealistically old age estimates for the deeper nodes in the tree. This is well illustrated by our analyses of the early hymenopteran radiation, where accounting for diversified sampling of extant taxa results in a major shift in the age estimate of Hymenoptera toward more recent times. Interestingly, the new estimates that accommodate the sampling bias remove much of the misfit observed previously between molecular divergence-time estimates and the fossil record. The diversity-sampled piecewise-constant FBD model on which these results are based provides the best a priori fit to our data, at least among the models examined here, and it shows all the signs of a well-behaved model in inference, including the ability to pick up relevant signal that is not detected by simpler models. Future studies will have to show to what extent this model is equally successful also in other dating analyses.

Taken together, this study and that of Ronquist et al. (2012a) highlight two important sources of error that can affect divergence time estimates in a major way: imperfect relaxed-clock models and a failure to accommodate the effect of sampling biases on the expected tree shape. Neither of them is unique to total-evidence dating; both are equally relevant to node dating. In our analyses, both these errors have tended to push the estimate of the crown age of Hymenoptera toward older time intervals. However, they could also bias age estimates in the other direction. For instance, even in our analyses, the failure to account for diversified sampling apparently causes many of the younger splits in the hymenopteran tree to be estimated too young (compare Figs. 6 and 7). Because of these complexities and the many other sources of error involved in divergence-time estimation, we refrain from speculation on how imperfect relaxed-clock models and failure to model sampling biases might have affected previous dating studies. Regardless of potential problems with past analyses, it is clear that total-evidence dating provides an ideal platform for going forward in exploring and further improving the models used for Bayesian divergence-time estimation. The processes of speciation, extinction, and fossilization are the real-world evolutionary mechanisms that affect present-day diversity and the observation of lineages as fossils through time. Thus, incorporating parameters that account for these processes is obviously important for epistemological reasons. From an empirical perspective, perhaps the most important aspect of the total-evidence approach is that it provides a common analytical platform, helping neontologists and paleontologists to include more information from the fossil record in future dating studies.

## Software availablity

The FBD priors are implemented in version 3.2.6 and newer of MrBayes (http://mrbayes.net; last accessed October 30, 2015). The commands are listed in the Appendix and in Zhang (2015).

## Supplementary Material

Supplementary tables and figures related to this article have been deposited at Dryad under http://dx.doi.org/10.5061/dryad.26820.

## Appendix

### MrBayes Commands

[calibrate the fossils]

calibrate Aulisca = unif(152,163)

 Sogutia = unif(174,201);

prset nodeagepr = calibrated;

[enforce the prior after calibrating]

prset brlenspr = clock:fossilization;

[net diversification, lambda-mu]

prset speciationpr = exp(100);

[turnover, mu/lambda]

prset extinctionpr = beta(1,1);

[fossil sampling proportion, psi/(mu + psi)]\ prset fossilizationpr = beta(1,1);

[extant sampling proportion, rho]

prset sampleprob = 0.0005;

[sampling strategy for extant taxa]

prset samplestrat = diversity [random];

[sampling strategy with rate shifting

 times #: t1 rho1, t2 rho2]

prset samplestrat = random 2: 252 0, 66 0;

prset samplestrat = diversity 1: 252 0;

[prior for tmrca]

prset treeagepr = offsetexp(315,396);

[relaxed-clock model]

prset clockvarpr=igr;

prset clockratepr=lognorm(-7.1,0.5);

[use ‘help prset’ for more info]
